# Early social distancing policies in Europe, changes in mobility & COVID-19 case trajectories: Insights from Spring 2020

**DOI:** 10.1371/journal.pone.0253071

**Published:** 2021-06-30

**Authors:** Liana R. Woskie, Jonathan Hennessy, Valeria Espinosa, Thomas C. Tsai, Swapnil Vispute, Benjamin H. Jacobson, Ciro Cattuto, Laetitia Gauvin, Michele Tizzoni, Alex Fabrikant, Krishna Gadepalli, Adam Boulanger, Adam Pearce, Chaitanya Kamath, Arran Schlosberg, Charlotte Stanton, Shailesh Bavadekar, Matthew Abueg, Michael Hogue, Andrew Oplinger, Katherine Chou, Greg Corrado, Tomer Shekel, Ashish K. Jha, Gregory A. Wellenius, Evgeniy Gabrilovich

**Affiliations:** 1 Department of Health Policy, London School of Economics, London, United Kingdom; 2 Google, LLC, Mountain View, CA, United States of America; 3 Department of Surgery, Brigham and Women’s Hospital and Harvard Medical School, Boston, MA, United States of America; 4 Department of Health Policy and Management, Harvard T.H. Chan School of Public Health, Boston, MA, United States of America; 5 University of Turin, Turin, Italy; 6 ISI Foundation, Turin, Italy; 7 Brown University School of Public Health, Providence, RI, United States of America; 8 Department of Environmental Health, Boston University School of Public Health, Boston, MA, United States of America; The Bucharest University of Economic Studies, ROMANIA

## Abstract

**Background:**

Social distancing have been widely used to mitigate community spread of SARS-CoV-2. We sought to quantify the impact of COVID-19 social distancing policies across 27 European counties in spring 2020 on population mobility and the subsequent trajectory of disease.

**Methods:**

We obtained data on national social distancing policies from the Oxford COVID-19 Government Response Tracker and aggregated and anonymized mobility data from Google. We used a pre-post comparison and two linear mixed-effects models to first assess the relationship between implementation of national policies and observed changes in mobility, and then to assess the relationship between changes in mobility and rates of COVID-19 infections in subsequent weeks.

**Results:**

Compared to a pre-COVID baseline, Spain saw the largest decrease in aggregate population mobility (~70%), as measured by the time spent away from residence, while Sweden saw the smallest decrease (~20%). The largest declines in mobility were associated with mandatory stay-at-home orders, followed by mandatory workplace closures, school closures, and non-mandatory workplace closures. While mandatory shelter-in-place orders were associated with 16.7% less mobility (95% CI: -23.7% to -9.7%), non-mandatory orders were only associated with an 8.4% decrease (95% CI: -14.9% to -1.8%). Large-gathering bans were associated with the smallest change in mobility compared with other policy types. Changes in mobility were in turn associated with changes in COVID-19 case growth. For example, a 10% decrease in time spent away from places of residence was associated with 11.8% (95% CI: 3.8%, 19.1%) fewer new COVID-19 cases.

**Discussion:**

This comprehensive evaluation across Europe suggests that mandatory stay-at-home orders and workplace closures had the largest impacts on population mobility and subsequent COVID-19 cases at the onset of the pandemic. With a better understanding of policies’ relative performance, countries can more effectively invest in, and target, early nonpharmacological interventions.

## Introduction

Non-pharmacological policies aimed at improving physical distancing, such as shelter in place orders or gathering bans, have been widely used to mitigate the spread of airborne pathogens, particularly in the early stages of a pandemic. While lockdowns have been utilized throughout the course of the COVID-19 pandemic, they are primarily intended to decrease the effective reproduction number (R_0_) to provide the time and bandwidth for public officials and the health system to enact other containment measures, such as comprehensive testing, tracing, and supportive isolation. However, as demonstrated by the COVID-19 pandemic, measures such as lockdowns are difficult to sustain in the long-term. There are often significant social and economic costs associated with social distancing policies [1, 2]. In this context, understanding the effectiveness of different types of policies employed during the first wave of the pandemic can be used to inform the decisions of policymakers weighing the costs of future lockdowns against the need to better mitigate the early spread of pandemic disease.

Prior work has shown the effect of social distancing orders on decreasing mobility and case growth in the US and India [3–7], but Europe remains a unique case study for the evaluation of early non-pharmacologic interventions [8, 9]. First, several European countries including Italy, Spain, and France were early hotspots of the COVID-19, hence more data is available to evaluate the first phase of the pandemic response [10, 11]. Second, while earlier studies have assessed the impact of social distancing policies, understanding the mechanisms through which social distancing policies may work—such as by reducing overall aggregated population-level mobility—would provide critical validation of public health interventions [8, 12]. Lastly, although not unique to Europe, understanding the relative effectiveness of mandatory vs. non-mandatory social distancing measures would enable public health officials across Europe and elsewhere to better implement early pandemic mitigation strategies in the future.

Quantifying the links between implementation of social distancing policies, decreases in mobility, and subsequent declines in COVID-19 cases is therefore critically important for policymakers in managing COVID-19, and may also inform the management of future emerging pandemics. Using a comprehensive COVID-19 policy database and aggregated mobility data linked to national counts of COVID-19 cases, we adopted a three-pronged strategy and sought to answer the following questions. First, to what extent did mobility decrease in response to social distancing policies across the study countries? Second, what type of policies (school closures, workplace closures, gathering bans or stay at home), and policy implementations (mandatory or non-mandatory) were most effective in decreasing mobility? And, finally, what was the relationship between the change in mobility and the trajectory of COVID-19 infections during the first wave of the pandemic?

## Materials and methods

### Data sources

This retrospective, country-level observational study uses data from three sources. First, we obtained data on national social distancing policies from the Oxford COVID-19 Government Response Tracker (OxCGRT), which has collected and aggregated information on the timing and nature of several specific government policy responses to the pandemic [13]. The data have been made publicly available and the methods of data collection and classification have been described elsewhere [6]. From these data, we considered the date of implementation at a national level of the following restrictions: school closures, workplace closures, cancellation of public events, and restrictions on internal movement such as shelter-in-place or stay-at-home orders (**S1 Table in S1 File**). Policies were further classified as mandatory or non-mandatory.

We used daily aggregated and anonymized data from tens of millions mobile device users in Europe who have turned on the Location History setting. The anonymized and aggregated dataset analyzed herein was the same one that was used to create the publicly-available Google COVID-19 Community Mobility Reports (first published at http://google.com/covid19/mobility on April 2, 2020). The data analyzed in this paper consisted of anonymized, aggregated, and differentially private counts of visits to places in different categories; the publicly available data reflects ratios computed using these counts.

Our analytic sample included 13,770 country-day observations across 27 European countries from January 3rd through April 19, 2020. Post-intervention data, with dates varying by country, represent relative mobility compared to a pre-COVID baseline for the same day of the week in the same location (computed over the period of 2020-01-03 through 2020-02-06). The methods for generating, aggregating, and anonymizing data were the same as those used in the creation of the publicly-available Google COVID-19 Community Mobility Reports [14, 15]. As in our prior work [6], we used relative change in the average number of hours spent away from places of residence as our primary mobility outcome. This metric is estimated as 24 (hours) minus the population-averaged number of hours spent at places of residence, divided by the corresponding number from the baseline period. We used publicly accessible mobility variables, including the number of visits to workplaces, grocery stores and pharmacies; retail stores, recreational sites, and eateries; transit stops; and parks were used as secondary outcomes. The anonymization process for these data was designed to ensure that no personal data, including an individual’s location, movement, or contacts, can be derived from the resulting metrics [14].

Finally, we obtained data on known COVID-19 cases from the European Centre for Disease Prevention and Control and national sources aggregated and standardized through the Center for Systems Science and Engineering (CSSE) at Johns Hopkins University [16].

### Policy identification

In Europe, large-scale restrictions on businesses and people’s movements with the intention of promoting social distancing were first enacted in Northern Italy in late February, 2020. Other European countries subsequently enacted a variety of mandatory and non-mandatory social distancing measures, including closures of schools, closing workplaces, cancelation of public events and restrictions on internal movement or stay at home policies. We reviewed all European countries, and we selected a subset of 27 study countries based on the availability of data on both social distancing orders and on population mobility. For each study country, we defined index dates based on the implementation of the first social distancing policy. We then compared the value of several mobility metrics in the week following the index date versus the 7-day period extending from 9 to 2 days prior to the index date. We included a two-day washout period prior to the index date given the volatility due to public messaging that typically precedes implementation of social distancing orders. Because each country is compared to itself, these estimates control for possible confounding by country-specific factors that remain relatively stable over this time period.

### Statistical analysis

We employed an interrupted time series design and linear mixed-effects models to first assess the relationship between policies and observed changes in weekly average mobility, and then to assess the relationship between changes in mobility and rates of COVID-19 infections in subsequent weeks.

#### Policy type & changes in mobility

We first assessed which policies were most effective in reducing average population mobility. We evaluated the association between enactment of national policies intended to foster social distancing and changes in population mobility. We applied a single interrupted time series approach using each country’s recent past as its own control (**S1 Fig in S1 File**). We next fit a linear mixed-effects model to quantify the impact of different social distancing policies on the relative change in time spent away from places of residence, accounting for within-country correlation (**S1 Appendix in S1 File**). Sensitivity analyses suggest these results were robust to alternative modeling choices (**S2 Table in S1 File**).

#### Changes in mobility & infections

We next used a linear mixed-effects model to assess the association between changes in mobility and subsequent changes in the number of new COVID-19 cases, a primary driver of policy decisions during this time. Specifically, we estimated the change in the log of new cases from one week to the next in each country as a function of recent weekly mobility changes. We modeled the country-specific time course of the pandemic by including an indicator variable for the first week the country reported 10 new cases and a separate variable to account for the number of weeks elapsed since then. We initially considered the impacts on case growth rates from changes in mobility in the prior 1, 2, 3, and 4 weeks. Using a forward selection approach (see **S1 Appendix in S1 File**), our final model only included mobility changes two weeks prior to the present week.

We report the results from this analysis as the change in case growth rate associated with a 10% and 50% decrease in population mobility. To further facilitate interpretation, we also present results as the difference over time in new and total COVID-19 cases given the actual observed changes in mobility versus the counterfactual scenario of no change in mobility (**S2 Fig in S1 File**). Confidence intervals for these estimates were estimated using a cluster bootstrap approach with the country as the cluster, as described in the **S1 File**.

## Results

**Fig 1:**
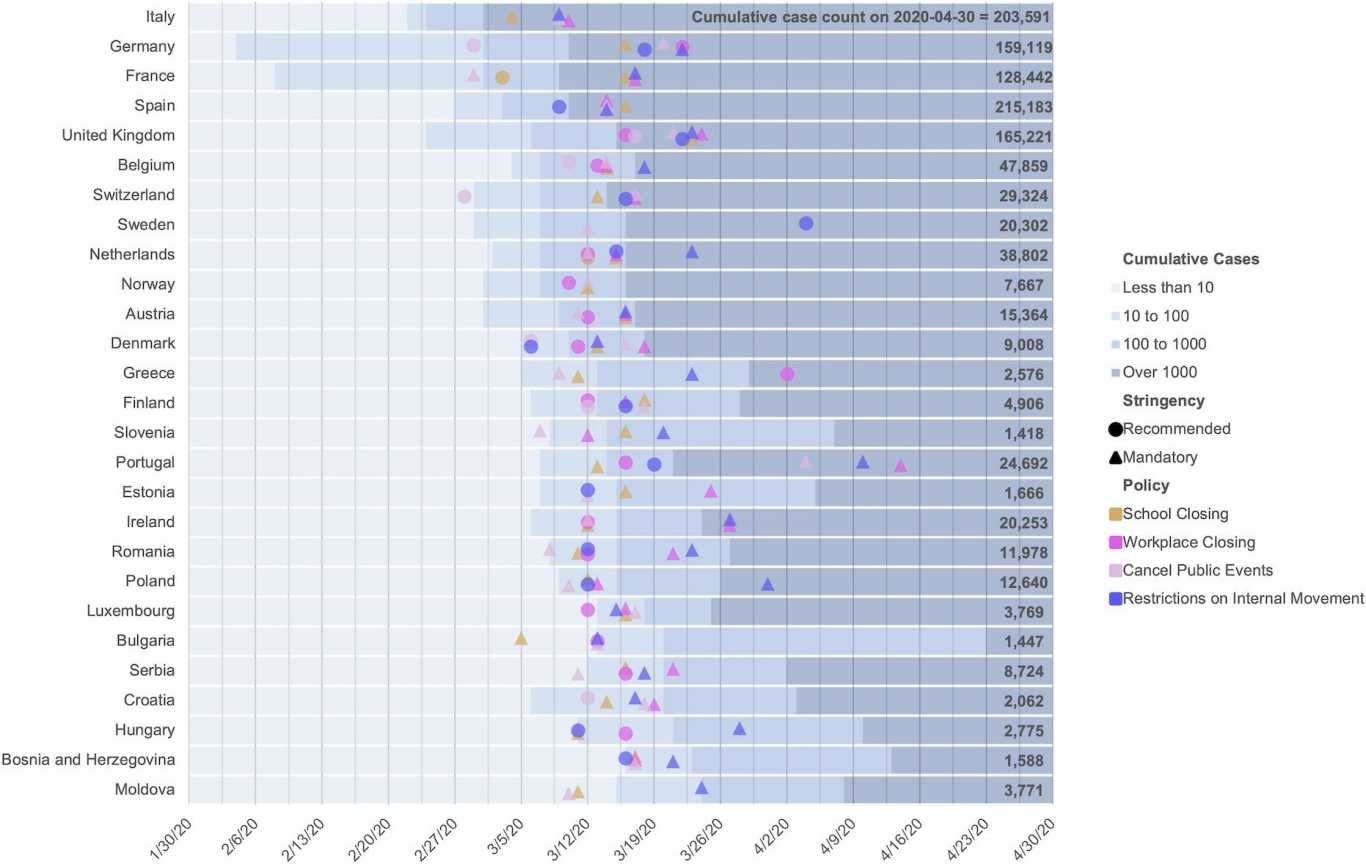
Timeline of major COVID-19 policy interventions by country. In response to the COVID-19 pandemic, European countries enacted a series of national policies intended to promote social distancing that varied across countries in terms of time (with respect to both calendar time and reported cases), nature, and enforcement (mandatory versus non-mandatory). Policy type is indicated with color and if that policy was instituted as a mandatory or non-mandatory policy is indicated with shape.

We found that national policies intended to promote social distancing and decrease community mobility were implemented within a relatively short time frame across Europe (**Fig 1**). Switzerland was the first country to institute a social distancing policy: a non-mandatory public gathering ban. This policy was followed by a clustering of closures implemented between March 12^th^ and 19^th^, with significant heterogeneity in policies across the European countries. Policies centered on the cancellation of public events largely preceded school closures, workplace closures and stay-at-home policies or restrictions on internal movement, though there was variation between countries in terms of the ordering of these policies. Policies also varied in terms of timing (with respect to both calendar time and time from reporting of the 10th, 100th and 1,000th cases), type, and implementation (mandatory versus non-mandatory). While Germany, France, the UK and other countries reported their first cases in February, the majority of national social distancing policies were implemented after a country first reported 10–100 cases.

**Fig 2:**
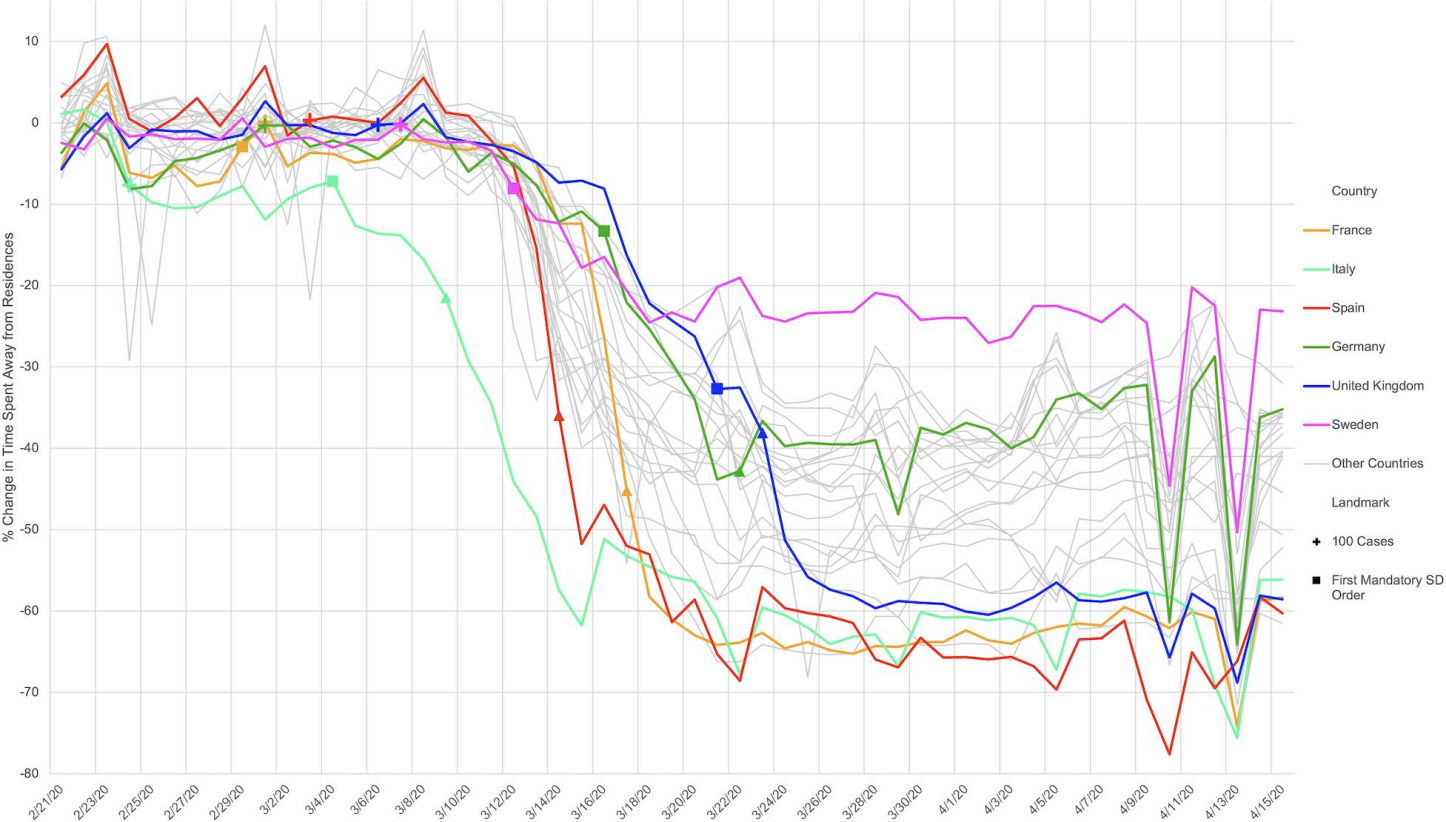
Relative change in mobility across study countries, Spring 2020. Relative change in mobility in European study countries, as estimated using aggregated (anonymized with differential privacy) data from Google users who have opted-in to Location History. For each country, crosses indicate the date on which the 100th case was reported, squares indicate the date of the first mandatory social distancing policy, and triangles denote the date of shelter-in-place orders.

Across the study countries social distancing policies had a substantial yet heterogeneous impact on population mobility. Italy was the first country to exhibit a notable decline in mobility, which began during the week of March 1^st^ [17]. Spain saw the largest mobility decrease from the pre-COVID baseline, as measured by the time spent away from residence, with a decline in aggregate mobility of nearly 70% (**Fig 2**). Sweden had the smallest decrease in mobility, with approximately a 20% decline from baseline at the end of the study period (April 12^th^ 2020). In certain countries (e.g., Italy), the initial observed decline in mobility preceded mandatory stay-at-home policies at the regional level [17], suggesting voluntary reductions in mobility as previously documented in the US [18].

When assessing the relationship between social distancing policies and changes in mobility, we found that mandatory policies were more effective than non-mandatory policies in reducing mobility (**Table 1**). For example, mandatory workplace closings were associated with a 13.3% reduction in mobility (95% CI: -20.5% to -6.1%), versus 11.2% for non-mandatory workplace closure policies (95% CI: -17.9% to -4.6%). On the other hand, mandatory stay-at-home policies or shelter-in-place orders were associated with 16.7% less mobility (95% CI: -23.7% to -9.7%) versus only 8.4% less mobility (95% CI: -14.9% to -1.8%) for non-mandatory stay-at-home policies. Non-mandatory large gathering bans were not associated with a statistically significant change in mobility. School closures were associated with a -13.0% (95% CI: -18.9% to -7.2%) change in mobility. Mandatory stay-at-home policies were associated with the largest change in mobility out of all policy types studied.

**Table 1:** Relationship between social distancing policies and changes in mobility.

Policy	Percent Point Change
	Spent Away from Residence (95% CI)
**School closures (S1)**	-13.0 (-18.9, -7.2)****
**Workplace closures (S2)**	
** Non-Mandatory**	-11.2 (-17.9, -4.6)***
** Mandatory**	-13.3 (-20.5, -6.1)****
**Limits on large gatherings and events (S3)**	
** Non-Mandatory**	**-0.5 (-7.7, 8.7)**
** Mandatory**	-7.8 (-14.0, -1.6)**
**Stay at home policies (S6)**	
** Non-Mandatory**	-8.4 (-14.9, -1.8)**
** Mandatory**	-16.7 (-23.7, -9.7)****
**Fixed effects R**^**2**^	**51.7%**

For school closures, all but one country mandated school closures so mandatory versus non-mandatory closures were not considered separately. Additional variations of the model can be found in **S3 Table in S1 File**.

*p < 0.10

**p < 0.05

***p < 0.01

****p < 0.001.

**Fig 3:**
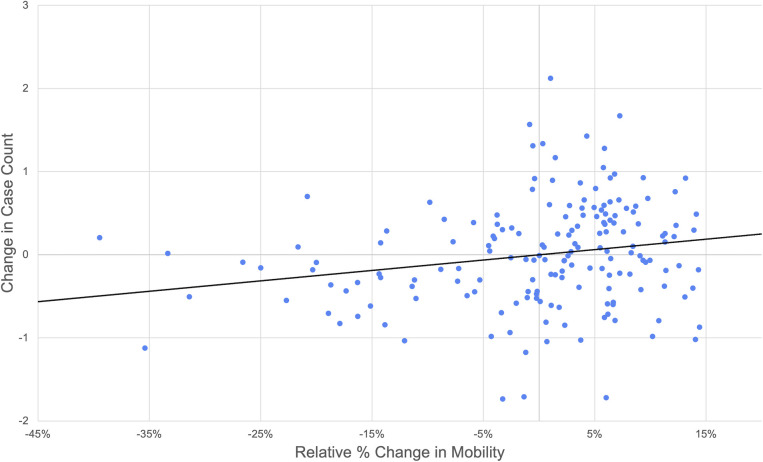
Linear relationship between relative change in mobility and change in case count. Partial regression plot of the 2-week-lagged change in mobility showing the relationship between mobility and case counts. Note higher variation in the response given small changes in mobility. The x-axis represents residuals in the relative percent change in mobility and the y-axis represents residuals in the case count.

Using a linear mixed effects model, we found a strong link between changes in mobility and changes in COVID-19 case growth (**S2 Fig in S1 File**). We estimated that decreases in mobility were approximately linearly related with subsequent change in case growth (**Fig 3**). For example, a 10% decrease in mobility was associated with an 11.8% decrease in new cases (95% CI: 3.8%, 19.1%) two weeks later. Furthermore, a more pronounced 50% decrease in mobility resulted in a 46.6% decrease in cases two weeks later (95% CI: 17.5%, 65.4%).

## Discussion

Across 27 European countries, we found that social distancing policies imposed early in the pandemic had substantial impacts on population mobility, but that the magnitude of this impact varied across policies and countries. We also observed a near 1:1 linear relationship between decreases in mobility and relative decline in COVID-19 case growth rate. These data provide important information relevant to understanding the impact of the initial lockdowns and other policies intended to limit the extent of the pandemic. For example, a 10% decrease in mobility was associated with an approximately 12% decrease in the number of newly reported cases two weeks later, highlighting the potential for aggregated mobility data to serve as a leading indicator of subsequent case trajectory. The largest reductions in mobility were associated with mandatory workplace closures and mandatory stay at home policies. Policies that involved mandated orders appear more effective in reducing time spent away from the residence compared to non-mandatory or recommended measures, with workplace closings and shelter-in-place-like orders being associated with the largest changes in mobility (each leading to approximately a 15% reduction in mobility the week after they are imposed).

Our findings have important implications for policy makers hoping to mitigate harm from the early pandemic spread of airborne pathogens. Policy makers combatting future waves of COVID-19 may be hesitant to return to strategies employed in the spring of 2020. Indeed, lockdowns and other extreme restrictions–particularly when mandated–cannot be sustained for long periods of time. These restrictions are intended to give time for countries to reduce the incidence of disease and put in place robust, and more sustainable, measures that prevent and control transmission. In line with this, comprehensive measures to find, test, trace, isolate, and support individuals who may be impacted by COVID-19 are integral and complementary to the policies we examine here. In this context, our findings are particularly relevant for early disease spread, and suggest that mandated measures were more effective in decreasing mobility and case growth as compared to voluntary measures during the first phase of the COVID-19 pandemic. For later stage mitigation, policymakers may need to consider intermittent cycles of mandatory social distancing to suppress the effective reproduction number (R_0_), allow for testing and tracing while also combatting the fatigue associated with such orders.

Ensuring policy choices are evidence-based is of critical importance when faced with high “lockdown fatigue”. As our results demonstrate, based on studying the effect of policies employed at the onset of the pandemic, decreasing mobility led to a nearly 1:1 reduction in the COVID-19 case count. Even with an effective vaccine, the roll out of life-saving pharmacological products can take years; many high need tuberculosis patients, for example, remain unvaccinated. In addition, our understanding of how specific airborne pathogens spread and in turn what constitutes a safe physical distance or the extent to which particulates remain on surfaces, improve with time. In line with this, risk mitigation strategies can be informed and improved by this knowledge, most notably during COVID the effectiveness of community masking. Indeed, mitigation strategies informed by new knowledge, such as mask wearing or improved ventilation systems in workplaces or schools [19], may mediate the relationship between social mobility and airborne infection during later lockdowns and social distancing measures, potentially requiring a smaller decrease in aggregate population mobility to achieve similar reductions in case growth.

While these results provide valuable new insights, governments must always consider their unique domestic and sub-national contexts. The consideration of school closures, for example, must be weighed with other country- or state-specific factors. This may include the share of the population that is food-insecure and receiving meals through the educational system or how much of the workforce would forgo employment in order to provide childcare at home. Particularly relevant within Europe’s cities are issues of safe and sustainable shared mobility, such as the need and ability of the population to utilize public transportation infrastructure [20]. In addition, how citizens view their health system has been shown to impact public response to early mitigation efforts. For example, Chan et al. found that regions with high trust in the government but low confidence in the healthcare system have higher adherence to social distancing policies [21]. They hypothesize that this may be attributable to citizens’ understanding that their health care system does not have adequate capacity but do trust the government represents their interests [21]. Regardless of baseline attributes, any single country’s policies, particularly those related to trade and travel restrictions, will impact its neighbors. The level of coordination of pandemic control policies across well-connected European countries can have large impacts on both travel and the continued community spread of disease [9, 22], but we were not able to factor these considerations into our analyses. There is considerable variability between and within-countries in terms of the degree of vulnerability of their populations, the trust these populations have in government measures and local governments’ ability to leverage resources for supportive, and safe, social distancing over time [23].

Our analysis adds to a growing literature aimed at assessing non-pharmaceutical interventions on COVID-19 in Europe by more closely examining the impact of social distancing policies [24]. In the spring of 2020, Dergiades et al. assessed the impact of government interventions across Europe on COVID-19 deaths [25]. Using daily data for 32 countries and examining the stringency of these policies, they found that stronger policy interventions (measured using the Oxford’s Government Response Tracker Index score) were more effective in slowing, or even reversing, the growth rate of COVID-19 deaths. By using real-time mobility data our work builds on this research and quantifies the policy strength in terms of how intensely people responded to the policy in practice, rather than the stringency of the policy as written. More recently, a pre-print by Oh et al. examined the impact of societal mobility restrictions across 36 countries [12]. Oh and colleagues looked at “commuting mobility,” which is an aggregate variable of visits to different types of community locations. Our work is similar, both in aim and countries studied, but examines relative time spent away from individuals’ primary place of residence—i.e. the amount of time people in a given location spend outside the home. Unlike mobility to and from specific locations, the average time that individuals spend away from their primary place of residence is significantly associated with COVID case growth.

### Limitations

This study has a number of limitations. First, countries do not implement policies at random. Observed differences may be confounded by country-level characteristics, such as varying degrees of public support for government policy more broadly. Moreover, there may be heterogeneous responses by populations of different countries to mandatory versus non-mandatory social distancing policies. As demonstrated by Bargain and Aminjonov, countries with higher baseline levels of government trust may not need to “enforce” a policy to achieve adherence [23]. However, during the time period we examine, we were not able to account for these country-level characteristics or other relevant factors, such as: variation in the underlying health-system, testing availability or adoption of corresponding non-pharmacological measures, such as masking or improved air filtration/ventilation of indoor spaces. Testing rates, in particular, have varied throughout the pandemic both between and within countries as well as over time, which likely constitutes a source of unmeasured bias. In addition, because social distancing policies and case milestones can occur in close proximity, causal attribution to one specific policy is challenging. As a result, our estimates represent the pooled effect of these events in practice, as opposed to the isolated response to a single action. In other words, we cannot exclude the possibility of residual confounding of within-country estimates by factors correlated with policy interventions or changes in mobility. Moreover, our analyses are limited to average country-level mobility during the study period, and therefore do not capture within-country heterogeneity. For instance, there was a significant diversity in response to social distancing policy at the sub-national level in both the US and India [6, 7].

Finally, we investigated the link between mobility and COVID-19 case growth rather than deaths. While COVID-19 mortality is an important outcome measure, we chose COVID-19 case growth as a primary outcome because mortality is a lagging indicator, sensitive to the demographic profile of each country, and sensitive to the health system response [26]. An additional limitation is that comparisons across countries using mobility data are descriptive. Since the Location History feature is off by default and requires explicit opt-in, countries may differ in the proportion of the population who opt-in to sharing Location History; the demographics of this group, the quality of the mobility data and of the Google Maps data about local establishments and other unobserved factors that may influence the observed changes in mobility. Finally, this study was limited to countries in which there were available data on both COVID-19 policies and mobility. As a result, the study does not include all countries in Europe.

## Conclusion

Our findings, based on analysis of spring 2020 data, offer actionable insights into which policies were most effective in decreasing aggregate mobility and infections during Europe’s first wave of the pandemic. With a better understanding of relative policy effectiveness at the beginning of the COVID-19 pandemic, countries can better design and target future non-pharmacological interventions based on what mitigated early spread of the disease.

## Supporting information

S1 File (PDF)

## References

[1] Mandel A, Veetil V. The Economic Cost of COVID Lockdowns: An Out-of-Equilibrium Analysis. Econ Disaster Clim Chang. 2020:1–21. doi: 10.1007/s41885-020-00066-z . 32838118 PMC7304379

[2] Thunström L, Newbold SC, Finnoff D, Ashworth M, Shogren JF. The Benefits and Costs of Using Social Distancing to Flatten the Curve for COVID-19. J Benefit Cost Anal. 2020:1–17. doi: 10.1017/bca.2020.12 PMC7242774.

[3] Auger KA, Shah SS, Richardson T, Hartley D, Hall M, Warniment A, et al. Association Between Statewide School Closure and COVID-19 Incidence and Mortality in the US. JAMA. 2020;324(9):859–70. doi: 10.1001/jama.2020.14348 32745200 PMC7391181

[4] Courtemanche C, Garuccio J, Le A, Pinkston J, Yelowitz A. Strong Social Distancing Measures In The United States Reduced The COVID-19 Growth Rate. Health Affairs. 2020;39(7):1237–46. doi: 10.1377/hlthaff.2020.00608 32407171

[5] Feyman Y, Bor J, Raifman J, Griffith KN. Effectiveness of COVID-19 shelter-in-place orders varied by state. PLOS ONE. 2021;15(12):e0245008. doi: 10.1371/journal.pone.0245008 33382849 PMC7775080

[6] Wellenius G, Vispute S, Espinosa V, Fabrikant A, Tsai T, Hennessy J, et al. Impacts of State-Level Policies on Social Distancing in the United States Using Aggregated Mobility Data during the COVID-19 Pandemic. arXiv [Internet]. April 2020 September 16, 2020. Available from: http://arxiv.org/abs/2004.10172.

[7] Woskie L, Tsai T, Wellenius G, Jha A. Early Impact of India’s Nationwide Lockdown on Aggregate Population Mobility and COVID-19 Cases. Lancet Pre-Print SSRN Electron J. 2020. doi: 10.2139/ssrn.3631258

[8] Flaxman S, Mishra S, Gandy A, Unwin HJT, Mellan TA, Coupland H, et al. Estimating the effects of non-pharmaceutical interventions on COVID-19 in Europe. Nature. 2020;584(7820):257–61. Epub 2020/06/09. doi: 10.1038/s41586-020-2405-7 . 32512579

[9] Ruktanonchai NW, Floyd JR, Lai S, Ruktanonchai CW, Sadilek A, Rente-Lourenco P, et al. Assessing the impact of coordinated COVID-19 exit strategies across Europe. Science. 2020;369(6510):1465–70. Epub 2020/07/19. doi: 10.1126/science.abc5096 ; PubMed Central PMCID: PMC7402626. 32680881 PMC7402626

[10] Oberhammer J. Social-distancing effectiveness tracking of the COVID-19 hotspot Stockholm. medRxiv. 2020:2020.06.30.20143487. doi: 10.1101/2020.06.30.20143487

[11] Pollán M, Pérez-Gómez B, Pastor-Barriuso R, Oteo J, Hernán MA, Pérez-Olmeda M, et al. Prevalence of SARS-CoV-2 in Spain (ENE-COVID): a nationwide, population-based seroepidemiological study. The Lancet. 2020;396(10250):535–44. doi: 10.1016/S0140-6736(20)31483-5 32645347 PMC7336131

[12] Oh J, Lee H-Y, Long KQ, Markuns JF, Bullen C, Artaza Barrios OE, et al. How well does societal mobility restriction help control the COVID-19 pandemic? Evidence from real-time evaluation. medRxiv. 2020:2020.10.29.20222414. doi: 10.1101/2020.10.29.20222414

[13] Oxford. OxCGRT. 2020.

[14] Aktay A, Bavadekar S, Coussoul G, Temp T, Temp A, Temp B, et al. Google COVID-19 Community Mobility Reports: Anonymization Process Description (version 1.0). arXiv [Internet]. April 2020 May 22, 2020. Available from: http://arxiv.org/abs/2004.04145.

[15] Google. COVID-19 Community Mobility Reports. 2020.

[16] Johns Hopkins Center for Systems Science and Engineering. CSSE COVID-19 Data Repository. 2020.

[17] Pepe E, Bajardi P, Gauvin L, Privitera F, Lake B, Cattuto C, et al. COVID-19 outbreak response, a dataset to assess mobility changes in Italy following national lockdown. Scientific Data. 2020;7(1):230. doi: 10.1038/s41597-020-00575-2 32641758 PMC7343837

[18] Badr HS, Du H, Marshall M, Dong E, Squire MM, Gardner LM. Association between mobility patterns and COVID-19 transmission in the USA: a mathematical modelling study. The Lancet Infectious Diseases. 2020;20(11):1247–54. doi: 10.1016/S1473-3099(20)30553-3 32621869 PMC7329287

[19] Brooks JT, Butler JC, Redfield RR. Universal Masking to Prevent SARS-CoV-2 Transmission—The Time Is Now. JAMA. 2020;324(7):635–7. doi: 10.1001/jama.2020.13107 32663243 PMC8607819

[20] Shokouhyar S, Shokoohyar S, Sobhani A, Gorizi AJ. Shared mobility in post-COVID era: New challenges and opportunities. Sustainable Cities and Society. 2021;67:102714. 10.1016/j.scs.2021.102714. PMC9760257 36569573

[21] Chan HF, Brumpton M, Macintyre A, Arapoc J, Savage DA, Skali A, et al. How confidence in health care systems affects mobility and compliance during the COVID-19 pandemic. PLOS ONE. 2020;15(10):e0240644. doi: 10.1371/journal.pone.0240644 33057450 PMC7561184

[22] Eckardt M, Kappner K, Wolf N. Covid-19 Acros European Regions: The Role of Border Controls. CEPR Discussion Paper No DP15178. August 2020.

[23] Bargain O, Aminjonov U. Trust and compliance to public health policies in times of COVID-19. Journal of public economics. 2020;192:104316–. Epub 2020/10/29. doi: 10.1016/j.jpubeco.2020.104316 . 33162621 PMC7598751

[24] Li Y, Campbell H, Kulkarni D, Harpur A, Nundy M, Wang X, et al. The temporal association of introducing and lifting non-pharmaceutical interventions with the time-varying reproduction number (R) of SARS-CoV-2: a modelling study across 131 countries. The Lancet Infectious Diseases. doi: 10.1016/S1473-3099(20)30785-4 33729915 PMC7581351

[25] Dergiades T, Milas C, Mossialos E, Panagiotidis T. Effectiveness of Government Policies in Response to the COVID-19 Outbreak. SSRN Electron J. May 2020. doi: 10.2139/ssrn.3602004 PMC10021334 36962226

[26] Ji Y, Ma Z, Peppelenbosch MP, Pan Q. Potential association between COVID-19 mortality and health-care resource availability. Lancet Glob Health. 2020;8(4):e480. Epub 2020/02/29. doi: 10.1016/S2214-109X(20)30068-1 ; PubMed Central PMCID: PMC7128131. 32109372 PMC7128131

