# Supplementary material for: Early social distancing policies in Europe, changes in mobility & COVID-19 case trajectories: Insights from Spring 2020
Source: PLoS One. 2021 Jun 30;16(6):e0253071. doi: 10.1371/journal.pone.0253071 (PMC8244916; doi:10.1371/journal.pone.0253071)

Supporting Information for:

Early Social Distancing Policies in Europe, Changes in Mobility & COVID-19 Case Trajectories: Insights from Spring 2020

Liana R. Woskie,[¶,1] Jonathan Hennessy, [¶,2] Valeria Espinosa, [¶,2] Thomas C. Tsai, [¶,3,8] Swapnil Vispute,[2] Benjamin H. Jacobson,[8] Ciro Cattuto,[4,5] Laetitia Gauvin,[5] Michele Tizzoni,[5] Alex Fabrikant,[2] Krishna Gadepalli,[2] Adam Boulanger,[2] Adam Pearce,[2] Chaitanya Kamath,[2] Arran Schlosberg,[2] Charlotte Stanton,[2] Shailesh Bavadekar,[2] Matthew Abueg,[2] Michael Hogue,[2] Andrew Oplinger,[2] Katherine Chou,[2] Greg Corrado,[2] Tomer Shekel,[2] Ashish K. Jha,[6] Gregory A. Wellenius,[2,7] Evgeniy Gabrilovich[*,2]

¶ = These authors contributed equally to this work.

* = corresponding author, gabr@google.com

Affiliations:

1. Department of Health Policy, London School of Economics, London, UK
2. Google, LLC. Mountain View, CA, USA
3. Department of Surgery, Brigham and Women's Hospital and Harvard Medical School, Boston, MA, USA
4. University of Turin, Turin, Italy
5. ISI Foundation, Turin, Italy
6. Brown University School of Public Health, Providence, RI, USA
7. Department of Environmental Health, Boston University School of Public Health, Boston, MA, USA
8. Department of Health Policy and Management, Harvard T.H. Chan School of Public Health, Boston, MA, USA

S1 Table: Policy Types, as Outlined in the Oxford COVID-19 Government Response Tracker (OxCGRT)

Policy	Description
**School Closures**	0 - No measures<br>1 - recommend closing<br>2 - Require closing (only some levels or categories, eg just high school, or just public schools)<br>3 - Require closing all levels
**Workplace Closures**	0 - No measures<br>1 - recommend closing (or work from home)<br>2 - require closing (or work from home) for some sectors or categories of workers<br>3 - require closing (or work from home) all-but-essential workplaces (e.g. grocery stores, doctors)
**Limits on Large Gatherings and Events**	0 - No restrictions<br>1 - Restrictions on very large gatherings (the limit is above 1000 people)<br>2 - Restrictions on gatherings between 101-1000 people<br>3 - Restrictions on gatherings between 11-100 people<br>4 - Restrictions on gatherings of 10 people or less
**Stay at Home Policies**	*Internal Movement*<br>0 - No measures<br>1 - Recommend not to travel between regions/cities<br>2 – Internal movement restrictions in place
	Stay at Home<br>0 - no measures<br>1 - recommend not leaving house<br>2 - require not leaving house with exceptions for daily exercise, grocery shopping, and 'essential' trips<br>3 - require not leaving house with minimal exceptions (eg allowed to leave once a week, or only one person can leave at a time, etc)

Listed policy types (workplace closures, etc.) are classified as mandatory or non-mandatory. The category “Stay at Home Policies” includes limits on internal movement, orders to "shelter-in-place" and otherwise confine to the home. This category was later updated to exclude shelter-in-place orders. As a result, we use the more inclusive term “stay at home policies” throughout this paper. Content was adapted from the Blavatnik School Working Paper, “Variation in Government Responses to COVID-19.” Also provided in the OxCGRT GitHub Repository, which can be accessed here: https://github.com/OxCGRT/covid-policy-tracker/blob/master/documentation/codebook.md

S1 Appendix. Extended Content on Methodology with Models

We first assessed which policies were most effective in reducing average population mobility. We evaluated the association between enactment of national policies intended to foster social/physical distancing and changes in population mobility. Specifically, we applied a single interrupted time series approach using each country's recent past as its own control. As in prior analyses, we designated the relative change in the average time spent away from places of residence as the primary outcome and the number of visits to workplaces, grocery stores and pharmacies; retail stores, recreational sites, and eateries; transit stops; and parks as secondary outcomes.

Specifically, we fit a linear mixed effects model where the outcome is the percent change in time spent away from residences between week j+1 (post) and week j-1 (pre) and including country as a random effect (i.e., $Country_i$) to account for within-country correlation. For week j, the covariates are determined by the social distancing orders issued during week j. That is, for country i and week j:

$$\left\{\frac{mobility_{j+1} - mobility_{j-1}}{mobility_{j-1}}\right\}_{ij} = \beta_0 + \sum_{s:\, social\ distancing\ order\ type} \beta_s \; I(s_{ij} = s) + Country_i + \epsilon_{ij}$$

(1)

We used a model that links social distancing policies and case milestones to changes in mobility at the country-week level. In these models, the response is the within-country change in mobility from week *j-1* to week *j + 1*and predictor variables describe policy and case changes in week *j* in the same country. In sensitivity analyses we additionally adjusted all models for the timing of reporting of the 10th, 100th, and 1000th cases in week *j* in order using the same coding scheme as above to account for the potential impact that increasing case counts in a given country may have had on public awareness of the pandemic. We found that the coefficients for the social distancing policies were robust to the inclusion of these case count milestones. The country random effect was not found to be significant, hence in Exhibit 3 and Supplementary Exhibit 3 we report the estimates for the fixed effects models.

We next assessed the association between changes in mobility and subsequent changes in the number of new COVID-19 cases. To do so, we fit a linear mixed effects model with the change in the log of new cases from one week to the next in a country as our response. The predictor variables included are the weekly mobility changes, the number of weeks since the country first hit 10 new cases in a week (to account for the progression of the pandemic), and a dummy variable for the first week the country hit 10 new cases (to mitigate potential noise resulting from changes in small case counts). Using a forward selection approach, our final model only included mobility changes two weeks prior to the present week. Specifically, the model we fit for week *i* and Country *j* (as a random effect) was:

$$ln\left\{\frac{new\ cases_{\ i+1}}{new\ cases_{\ i}}\right\}_j = \gamma_0 + \gamma_{week_0} I(week_0) + \gamma_{week} week_i + \sum_{lag:\,0,\dots,4} \gamma_{lag} \left\{\frac{mobility_{i-(lag+1)} - mobility_{i-lag}}{mobility_{i-lag}}\right\} + Country_j + \epsilon_{ij}$$

(2)

We sought to visualize these results in a more intuitive way in terms of the relative change in total case count progression. We compared the progression with mobility changes to the progression without mobility changes. The final exhibit shows the total and new case count relative change progression between these scenarios for the average country after exceeding 10 cases. To account for the uncertainty of these estimates we ran a cluster bootstrap analysis with the country as the cluster. More specifically, we sampled countries with replacement, refit model (2) on the sample and calculated the total and new case counts for the average country with and without the mobility changes. We repeated this 1,000 times and calculated the standard error for the percent changes of total and new case counts.

As summarized in the results section, we estimated that the relationship between case growth and mobility was close to linear. The coefficients were:

$$\gamma_0 = 2.1\ (95\%\ CI{:}\ 1.9, 2.4),\ \gamma_{week_0} = 1.0\ (95\%\ CI{:}\ 0.7, 1.3),\quad \gamma_{week} = -0.4\ (95\%\ CI{:}\ -0.5, -0.3)$$

country random effect variance = 0.085 (SE: 0.07)

S2 Table. Relationship Between Social Distancing Policies and Changes in Mobility

	Percentage point change in time spent away from places of residence (95% CI)		
Policy	**Model I** With no case counts	**Model II** With case counts	**Model III** Excluding countries driving the 1K case magnitude
School closures	**-13.0 (-18.9, -7.2)********	-11.9 (-17.4, -6.5)****	-15.0 (-20.6, -9.4)****
Workplace closures			
Non-Mandatory	**-11.2 (-17.9, -4.6)******	-11.2 (-13.1, 4.7)***	-9.0 (-15.4,- 2.6)***
Mandatory	**-13.3 (-20.5, -6.1)********	-14.1 (-19.9, -6.2)****	-12.3 (-19.3, -5.4)****
Limits on large gatherings/events			
Non-Mandatory	**-0.5 (-7.7, 8.7)**	-1.9 (-10.2, 6.5)	-4.6 (-13.2, 4.1)
Mandatory	**-7.8 (-14.0, -1.6)****	-9.1 (-17.2, -5.2)***	-9.7 (-15.8, -3.5)***
Stay at home policies			
Non-Mandatory	**-8.4 (-14.9, -1.8)****	-7.9 (-14.1, -1.7)**	-5.3 (-11.6, 1.0)
Mandatory	**-16.7 (-23.7, -9.7)********	-14.7 (-21.5, -7.9)****	-15.3 (-22.1, -8.4)****
10 reported cases		0.8 (-4.4, 6.1)	0.9 (-4.5, 6.1)
100 reported cases		-6.9 (-12.1, -1.7)**	-9.7 (-15.2, -4.3)***
1000 reported cases		-12.1 (-17.7, -6.5)****	-6.6 (-12.4,-0.8)**
Fixed effects R^2	51.7%	58.6%	63.0%

In Model III Germany, France, Netherlands & UK were excluded. Based on the timeline of these events, we believe that the way the weeks are split gives a higher attribution to the 1000 case count than it should because for all of these countries, important social distancing measures were imposed on the following week, inflating its attribution share. For school closures, only one country had a non-mandatory or “recommended” school closure. Thus, we did not disaggregate mandatory vs. non-mandatory school closures. *p < 0:10 , **p < 0:05 , ***p < 0:01 , ****p < 0:001.

S1 Figure. Relationship Between Social Distancing Policies and Changes in Mobility
Each panel depicts the change in mobility associated with a given intervention across all study countries. For example, in the first panel the "intervention" is a single date - the implementation of Italy's national lockdown. The second panel shows the average impact when each country reached 100 cases. The third and fourth panels show the impact of the first mandatory social distancing order and implementation of mandatory restrictions on internal movements, respectively. The dashed vertical line separates the primary from secondary metrics considered.

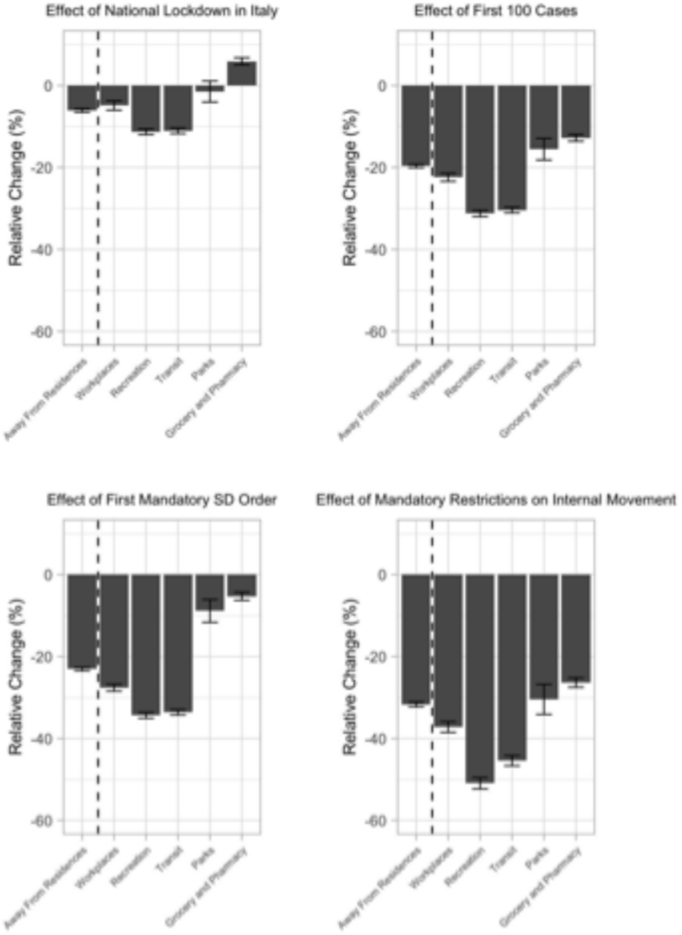

S2 Figure. Percent Change in Total Case Count and New Case Count Attributed to Mobility, Average Across Study Countries After Exceeding 10 Cases

Cluster bootstrap analysis, demonstrating the link between changes in mobility and changes in COVID-19 case growth. The line represents the average percent drop in total and new cases comparing the case count under the hypothetical scenario of no mobility change to the observed mobility change. For instance, the new case count in the fourth week after exceeding 10 cases is about 20% lower than it would have been had there been no mobility change.

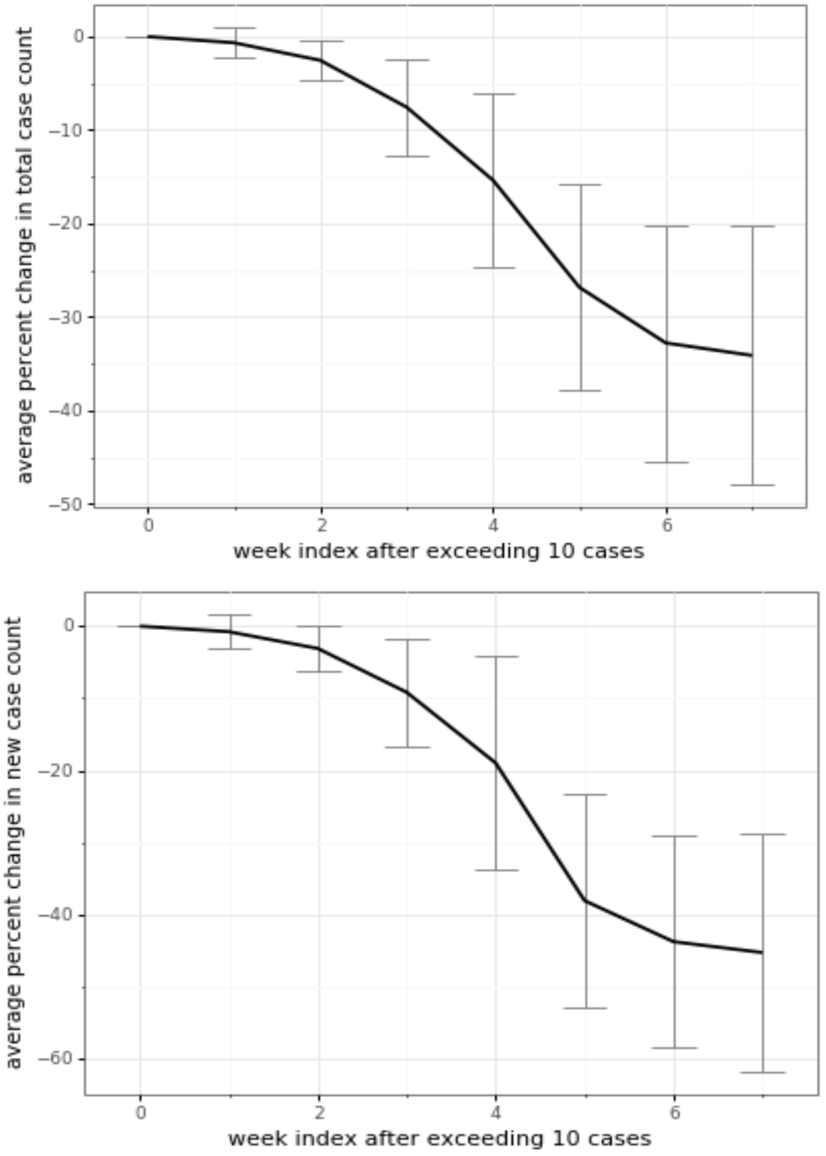

Supplement: S1 File — (PDF) [file pone.0253071.s001.pdf]
